# Toll-like receptor 9 expression is associated with breast cancer sensitivity to the growth inhibitory effects of bisphosphonates *in vitro* and *in vivo*

**DOI:** 10.18632/oncotarget.13570

**Published:** 2016-11-24

**Authors:** Jouko Sandholm, Jaakko Lehtimäki, Tamiko Ishizu, Sadanandan E. Velu, Jeremy Clark, Pirkko Härkönen, Arja Jukkola-Vuorinen, Aleksi Schrey, Kevin W. Harris, Johanna M. Tuomela, Katri S. Selander

**Affiliations:** ^1^ Cell Imaging Core, Turku Centre for Biotechnology, University of Turku and Åbo Akademi University, Turku, Finland; ^2^ Department of Cell Biology and Anatomy, University of Turku, Turku, Finland; ^3^ MediCity Research Laboratory/PET, Turku PET Centre, University of Turku, Turku, Finland; ^4^ Department of Chemistry, University of Alabama at Birmingham, Birmingham, AL, U.S.A; ^5^ Oulu University Hospital, Department of Oncology, Oulu, Finland; ^6^ Department of Otorhinolaryngology – Head and Neck Surgery, Turku University Hospital, Turku, Finland; ^7^ Department of Medicine, University of Alabama at Birmingham, Birmingham, AL, U.S.A; ^8^ Birmingham Veterans Affairs Medical Center, Birmingham, AL, U.S.A; ^9^ UAB Comprehensive Cancer Center, Birmingham, AL, U.S.A; ^10^ Department of Pathology, Lapland Central Hospital, Rovaniemi, Finland

**Keywords:** breast cancer, bisphosphonate, biomarker, TLR9, ApppI

## Abstract

Bisphosphonates are standard treatments for bone metastases. When given in the adjuvant setting, they reduce breast cancer mortality and recurrence in bone but only among post-menopausal patients. Optimal drug use would require biomarker-based patient selection. Such biomarkers are not yet in clinical use. Based on the similarities in inflammatory responses to bisphosphonates and Toll-like receptor (TLR) agonists, we hypothesized that TLR9 expression may affect bisphosphonate responses in cells. We compared bisphosphonate effects in breast cancer cell lines with low or high TLR9 expression. We discovered that cells with decreased TLR9 expression are significantly more sensitive to the growth-inhibitory effects of bisphosphonates *in vitro* and *in vivo*. Furthermore, cancer growth-promoting effects seen with some bisphosphonates in some control shRNA cells were not detected in TLR9 shRNA cells. These differences were not associated with inhibition of Rap1A prenylation or p38 phosphorylation, which are known markers for bisphosphonate activity. However, TLR9 shRNA cells exhibited increased sensitivity to ApppI, a metabolite that accumulates in cells after bisphosphonate treatment. We conclude that decreased TLR9-expression sensitizes breast cancer cells to the growth inhibitory effects of bisphosphonates. Our results suggest that TLR9 should be studied as a potential biomarker for adjuvant bisphosphonate sensitivity among breast cancer patients.

## INTRODUCTION

Bisphosphonates (BPs) are synthetic analogues of the naturally occurring pyrophosphate. These drugs inhibit osteoclast-mediated bone resorption and they have an established role in the treatment of bone conditions that involve increased osteoclast activity, such as postmenopausal or treatment-induced osteoporosis, multiple myeloma and skeletal metastases of solid tumors [1, 2]. In these settings, BPs are unequivocally beneficial as they inhibit hypercalcemia, bone pain and fractures. Apart from the rare side effects, which include oesophageal irritation, renal dysfunction, osteonecrosis of the jaws, and atypical sub-trochanteric femoral fractures, BPs are generally well tolerated [3, 4].

Depending on their molecular structure, these drugs are divided into pyrophosphate-resembling (p-BPs, such as clodronate and etidronate) and nitrogen-containing BPs (n-BPs, such as alendronate, pamidronate, risedronate and zoledronate) [5]. The newer n-BPs are more potent inhibitors of bone resorption, zoledronate being the most potent. The cellular mechanisms of action of BPs vary according to their molecular structure. P-BPs are metabolized into toxic, apoptosis-inducing ATP-analogs (AppCCl_2_) inside the cells. The primary mode of action of n-BPs is to inhibit the farnesyl pyrophosphate synthase of the mevalonate pathway, which is in the beginning of the cholesterol biosynthesis [5, 6]. This results in a decreased cellular pool of prenyl groups and leads to impaired post-transcriptional prenylation of small GTPases that are required for a great variety of cellular functions, such as vesicular transportation during the bone resorption phase of osteoclasts [5, 7]. In addition, although n-BPs are not metabolized into ATP-analogs, they induce intracellular formation of such an analog, ApppI [8].

BPs exhibit high affinity to bone matrix hydroxyapatite. This and the inhibition of vicious cycle between osteoclast-mediated bone resorption and tumor growth in bone are considered the primary mechanisms mediating their anti-tumor efficacy in bone metastases [9, 10]. However, unlike initially thought, it is now clear that n-BPs may have direct anti-tumor effects also in soft tissues [11–17]. These direct cancer growth inhibitory effects of n-BPs are also mediated via the mevalonate pathway and intracellular ApppI [17–20]. Discovery of the anti-tumor effects has shifted the focus of BP studies from treating bone metastases to their adjuvant use, especially in early breast cancer [21, 22]. Several large clinical adjuvant trials have been conducted by now, mostly with zoledronate, to address whether adjuvant BPs prevent relapses in bone or elsewhere and affect breast cancer mortality [23–26]. These studies have been conducted in unselected breast cancer patient populations and in many of them BPs were found to be beneficial only among post-menopausal women. A recent, large meta-analysis of these studies, consisting of 18206 patients indeed confirmed that only post-menopausal breast cancer patients benefit from adjuvant BPs. In this patient group, adjuvant BPs produced highly significant reductions in bone recurrence (RR 0.72, 95% CI 0.60–0.86; 2p=0.0002) and breast cancer mortality (RR 0.82, 0.73–0.93; 2p=0.002).These effects were independent of tumor parameters (estrogen receptor status or grade), nodal status, bisphosphonate class, or dosing schedule or concomitant chemotherapy [23, 26]. Taken together, these studies suggest that there is a subset of post-menopausal breast cancer patients who may greatly benefit from adjuvant BPs in terms of relapses and survival [26]. Identification of such patients based on tumor characteristics would allow optimal adjuvant BP use. Currently, however, there are no such biomarkers in clinical use.

Toll-like receptor 9 (TLR9) is an intracellular DNA-receptor that recognizes both microbial and host-derived DNA [27]. TLR9 activation initiates a rapid and a robust innate immune response, with increased secretion of inflammatory mediators [27]. We have previously demonstrated that TLR9 is widely expressed in breast cancers [28–30]. We also showed that tumor TLR9 expression is associated with prognosis, but only among patients that have triple-negative tumors for which there are no targeted therapies [30]. Interestingly, n-BPs induce a similar rapid inflammatory response as TLR-ligands in cells[31, 33]. Furthermore, n-BPs have been shown to potentiate the pro-inflammatory effects of TLR ligands in bone marrow- or peripheral blood-derived mononuclear cells [34]. Based on the similarities in the responses of TLR ligands and BPs, we hypothesized that TLR9 expression may affect cellular responses to BPs. We demonstrate here for the first time that decreased TLR9 expression sensitizes breast cancer cells to the growth inhibitory effects of both p- and n-BPs. Our results suggest that tumor TLR9 expression status should be investigated as a potential biomarker for adjuvant BP use in breast cancer.

## RESULTS

### Breast cancer cells with decreased TLR9 expression exhibit increased sensitivity to the growth inhibitory effects of BPs *in vitro*

**Figure 1:**
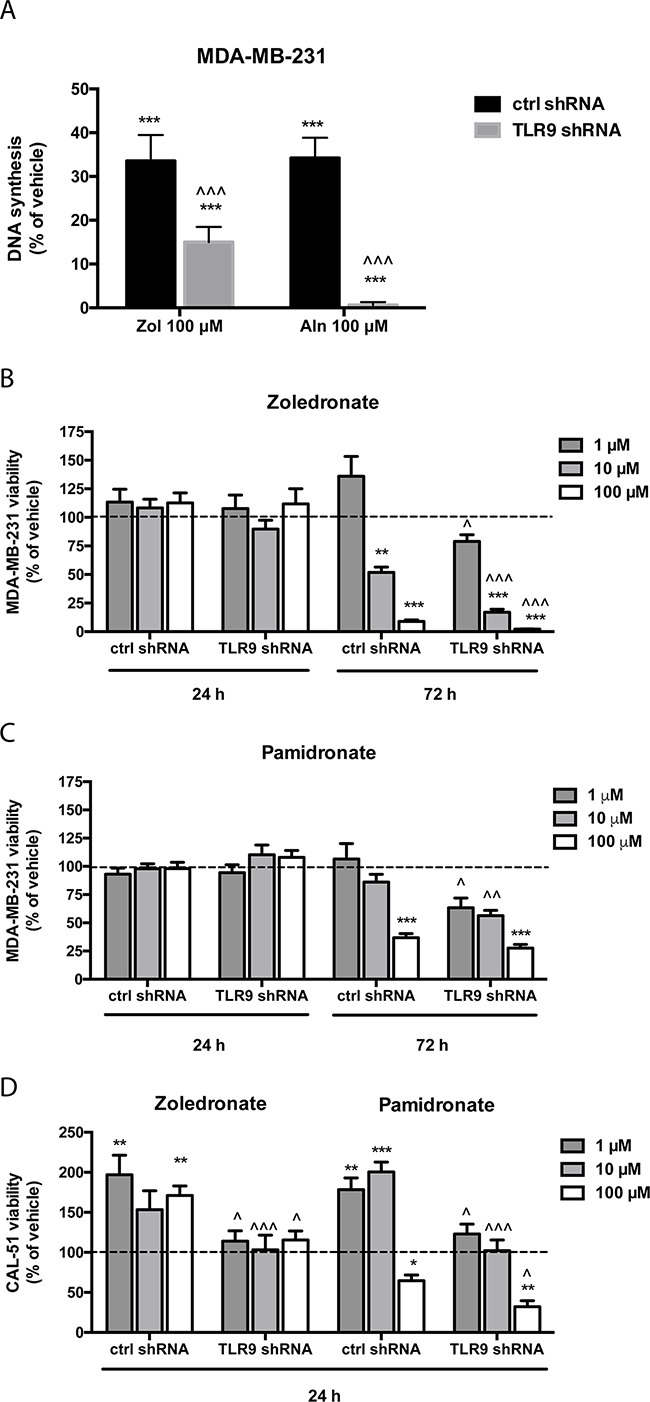
Cells with decreased TLR9 expression exhibit increased sensitivity to the growth inhibitory effects of n-bisphosphonates *in vitro* **A**. Human MDA-MB-231 breast cancer single cell clones stably expressing control shRNA or TLR9 shRNA plasmids were plated on 96-well plates. PBS as a vehicle control or 100 μM zoledronate (Zol) or alendronate (Aln) were added to the wells for 48 h, after which DNA synthesis was assessed with BrdU-assays. The bars represent proliferating cells, as a % of PBS-control. N=5, mean ± s.e.m, *** *p*<0.001 vs. vehicle, ^^^ *p* <0.001 vs. the corresponding control shRNA cells. **B**. MDA-MB-231 pools stably transfected with control shRNA or TLR9 shRNA-expressing plasmids were similarly cultured on 96-well plates for 24 or 72 h in the presence of 1- 100 μM zoledronate or **C**. pamidronate. **D**. CAL-51 cell pools stably expressing control or TLR9 shRNA were cultured for 24 h in the presence of vehicle or 1- 100 μM pamidronate or zoledronate. The bars represent cell viability as % of the corresponding vehicle treatment (dotted line). Mean ± s.e.m., n=6, ** *p* < 0.01, *** *p*< 0.001 vs. vehicle treatment, ^ *p*<0.05, ^^ *p*<0.01, ^^^ *p*<0.001 vs. corresponding control shRNA cells.

We initially studied the growth effects of n-BPs with single cell MDA-MB-231 clones that were stably transfected with a plasmid-based TLR9 shRNA or the corresponding empty vector [35]. The cells were cultured in the presence of vehicle or with 100 μM zoledronate or alendronate for 48 h, after which proliferation was measured with a BrdU-assay. Both BPs inhibited significantly the proliferation of all cells. The effect was, however, significantly more pronounced in the TLR9 shRNA cells than in the control shRNA cells (Figure 1A). To study this initial observation further, we did similar studies but now with pools of MDA-MB-231 and CAL-51 cells that had been stably transfected with control or TLR9 shRNA [30, 36]. The cells were treated with vehicle or 1-100 μM zoledronate or pamidronate. Viability was measured at 24 or 72 h time points with MTS assay. BPs did not affect the viability of either control or TLR9 shRNA MDA-MB-231 cells at 24 h. However, at 72 h both zoledronate and pamidronate induced a dose-dependent decrease in MDA-MB-231 cell viability. Depending slightly on the n-BP and concentration used, the effects were, however, significantly more pronounced in the TLR9 shRNA cells than in the control shRNA cells (Figures 1B – 1C). In CAL-51 cells, changes in viability were detected already after 24 h. Compared with the vehicle treatment, all studied zoledronate concentrations actually increased viability of the control shRNA CAL-51 cells, but not of the TLR9 shRNA CAL-51 cells. Pamidronate (100 μM) decreased significantly control shRNA CAL-51 cell viability. Also this effect was more pronounced in the TLR9 shRNA CAL-51 cells. Finally, the lower pamidronate concentrations (1 – 10 μM) induced a significant increase in control shRNA CAL-51 viability, but not in the TLR9 shRNA CAL-51 cells (Figure 1D).

**Figure 2:**
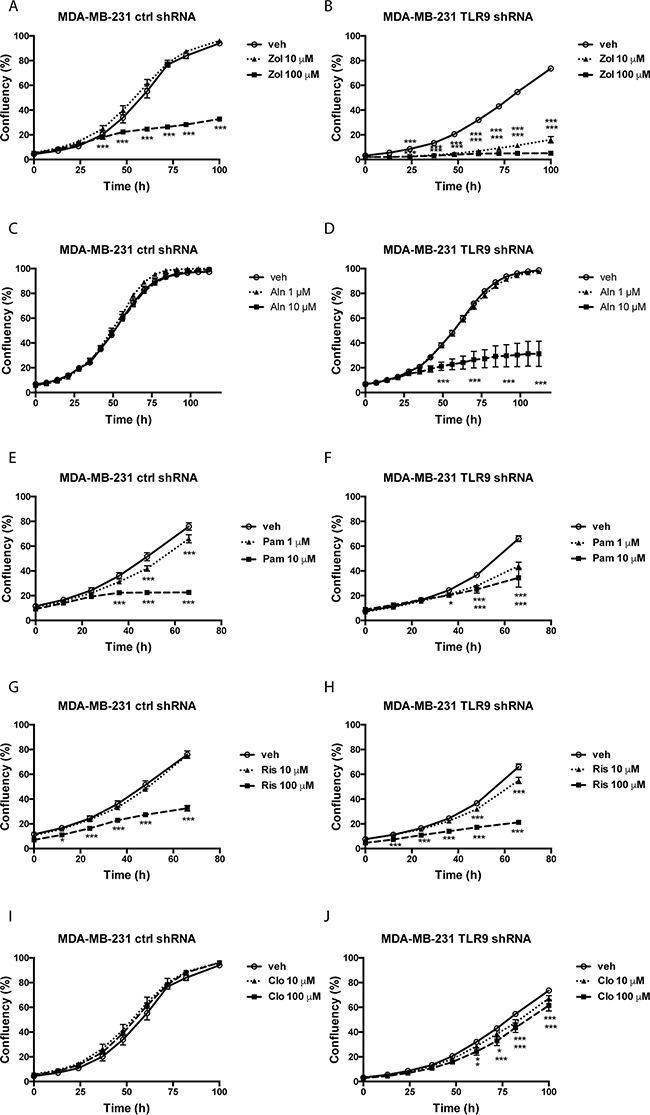
Control and TLR9 shRNA MDA-MB-231 cells exhibit differences in bisphosphonate-induced changes in cell confluency Control or TLR9 shRNA MDA-MB-231 cells were plated on 96-well plates in the presence of vehicle (veh) or indicated bisphosphonates (Zol = zoledronate, Aln = alendronate, Pam = pamidronate, Ris = risedronate, Clo = clodronate). Cell confluency was measured as a function of time by image analysis. Data is expressed as % confluency. Mean ± s.e.m, n = 3-8, * *p* <0.05, *** *p* < 0.001 vs. vehicle.

**Figure 3:**
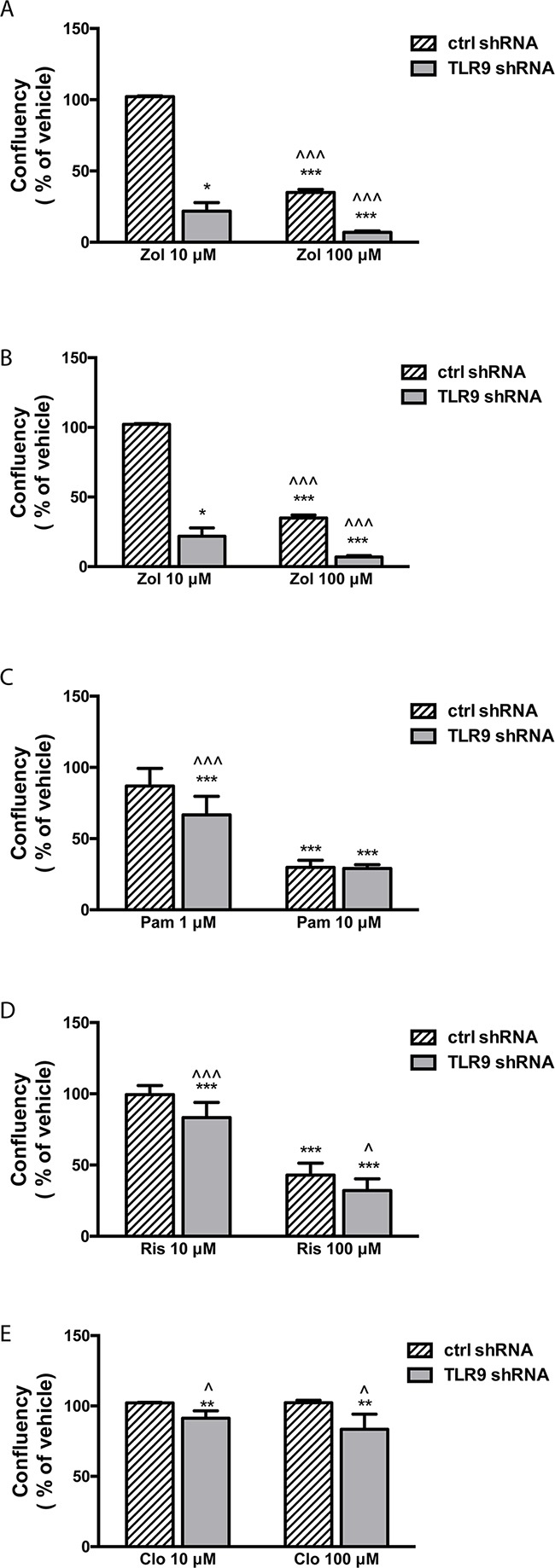
TLR9 shRNA MDA-MB-231 cells are more sensitive to the growth inhibitory effects of bisphosphonates than the corresponding control shRNA cells Confluency of cells was calculated as a % of vehicle and compared between control and TLR9 shRNA cells at the last timepoint of the growth curves (from Figure 2. Zol = zoledronate, Aln = alendronate, Pam = pamidronate, Ris = risedronate, Clo = clodronate). The bars represent mean ± s.d, n = 3-8, * *p*<0.05, *** *p* < 0.001 vs. corresponding vehicle, ^ *p* < 0.05, ^^^ *p*<0.001 vs. corresponding control shRNA cells.

Next, we measured the effects of BPs on cell growth as a function of cell confluency, using the IncuCyte set-up. Representative images of the vehicle- and zoledronate-treated MDA-MB-231 cells are shown in Supplementary Figure S2. Only the highest zoledronate concentration (100 μM) significantly inhibited the growth of control shRNA MDA-MB-231 cells, while 10 μM zoledronate had no effect (Figure 2A). On the contrary, both zoledronate concentrations significantly inhibited the growth of the TLR9 shRNA cells. Furthermore, the zoledronate-induced growth delays started to show earlier in the TLR9 shRNA cells (Figure 2B). Alendronate (1 or 10 μM) had no effect on the growth of control shRNA MDA-MB-231 cells (Figure 2C). In TLR9 shRNA cells, 10 μM alendronate was significantly growth inhibitory (Figure 2D). Also the growth inhibitory effects of pamidronate and risedronate were more pronounced in the TLR9 shRNA cells, in comparison to control shRNA cells (Figures 2E – 2H). Finally, similar differences, although weaker, were also detected between control and TLR9 shRNA MDA-MB-231 cells after clodronate treatment. In control shRNA cells, neither clodronate concentration affected growth. In the TLR9 shRNA cells, both clodronate concentrations induced a small but a significant growth inhibition (Figures 2I – 2J). The relative confluency of the control and TLR9 shRNA cells was compared at the final time point. Although the effect was slightly BP molecule- and concentration-dependent, BPs had a more profound growth inhibitory effect on TLR9 shRNA cells in all cases. The effect was most dramatic with 10 μM zoledronate (Figure 3).

**Figure 4:**
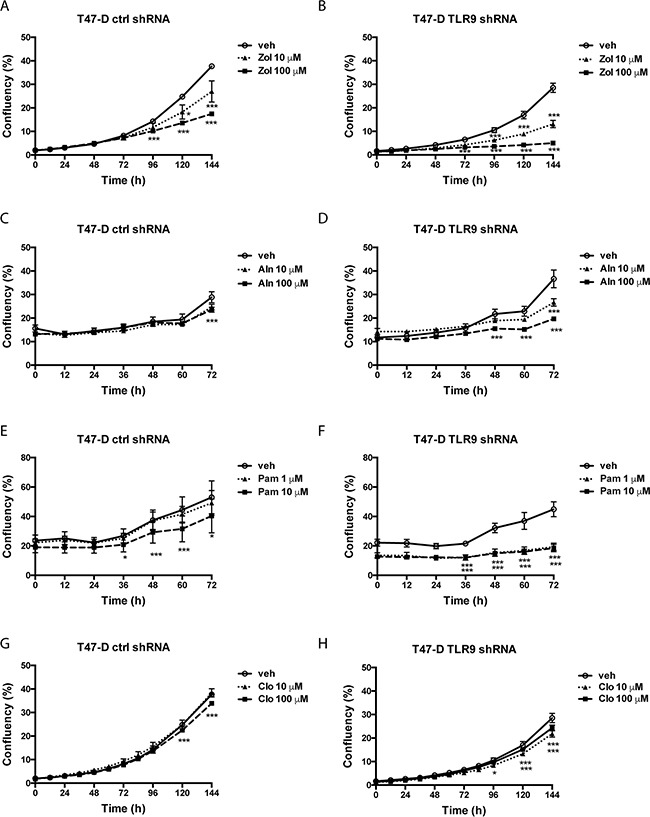
Control and TLR9 shRNA T47-D cells exhibit differences in bisphosphonate-induced changes in cell confluency Control and TLR9 shRNA T47-D cells were plated on 96-well plates in the presence of vehicle or the indicated bisphosphonates (Zol = zoledronate, Aln = alendronate, Pam = pamidronate, Clo = clodronate). Cell confluency was measured as a function of time by image analysis. Data is expressed as % confluency. Mean ± s.e.m, n = 3-4, * *p* <0.05, *** *p* < 0.001 vs. vehicle.

**Figure 5:**
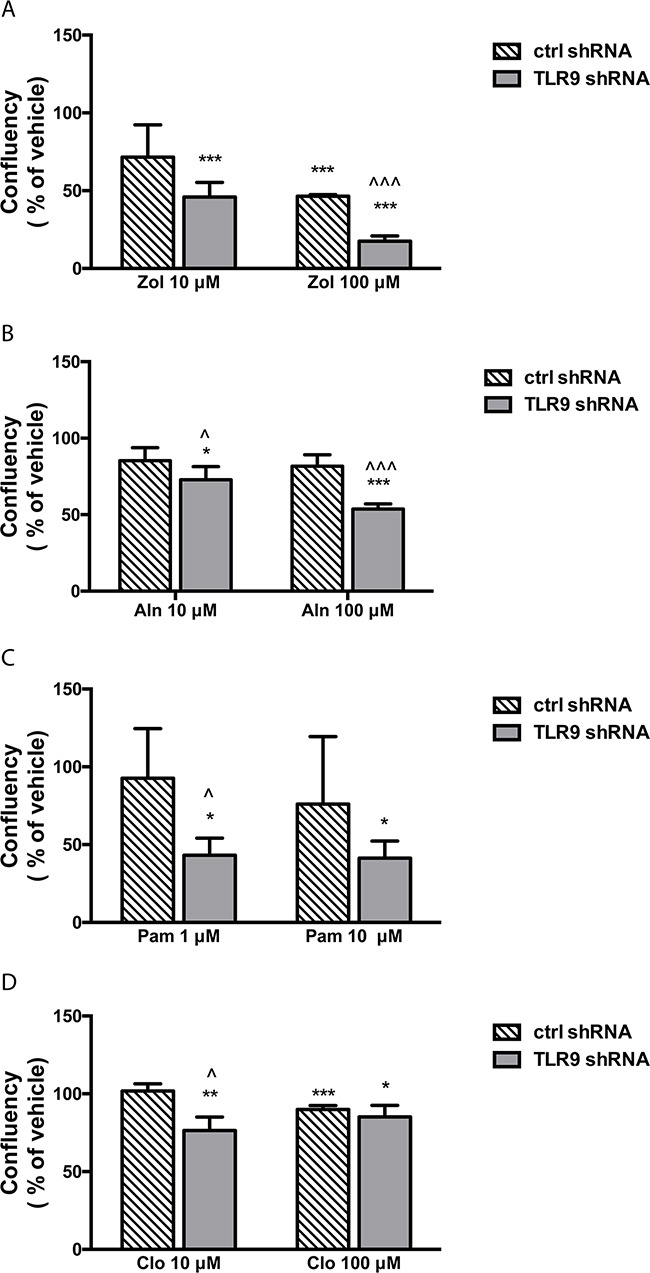
TLR9 shRNA T47-D cells are more sensitive to the growth inhibitory effects of bisphosphonates than the corresponding control shRNA cells Confluency of cells was calculated as a % of vehicle and compared between control and TLR9 shRNA cells at the last timepoint of the growth curves (from Figure 4. Zol = zoledronate, Aln = alendronate, Pam = pamidronate, Clo = clodronate). The bars represent mean ± s.d, n = 3-4, * *p*<0.05, *** *p* < 0.001 vs. corresponding vehicle, ^ *p* < 0.05, ^^^ *p*<0.001 vs. corresponding control shRNA cells.

We performed similar studies also with the ER-positive control and TLR9 shRNA T47-D cells. In essence, the results were similar to those seen in MDA-MB-231 cells. The growth of the T47-D TLR9 shRNA cells was inhibited with smaller BP concentrations and/or they exhibited a greater growth inhibitory response to BPs than the corresponding control shRNA cells (Figure 4). Again, although the effect was slightly BP molecule- and concentration-dependent, BPs had a more profound growth inhibitory effect in TLR9 shRNA cells in all cases. When the growth inhibition was compared at the final time point, the effect was most dramatic with 100 μM zoledronate (Figure 5). Similarly, both pamidronate and alendronate were stronger inducers of growth inhibition in TLR9 shRNA 4T1 mouse mammary carcinoma cells, as compared with the corresponding control shRNA cells (Supplementary Figure S3). Of the two BPs studied with the 4T1 cell line, 10 μM pamidronate had the most dramatic effect. Taken together, regardless of the differences between the BP concentrations and cell lines used, these results suggest that cells with decreased TLR9 expression are more sensitive to the growth inhibitory effects of BPs. The effect is greatest with n-BPs, but it is also detectable with clodronate. These results are in line with those observed with parental cell lines. MCF-7 cells, which express lower TLR9 protein levels than MDA-MB-231 or T47-D cells [29], are also the most sensitive to BPs (Supplementary Figure S4).

### Inhibition of Rap1A prenylation and p38 phosphorylation are independent of TLR9

**Figure 6:**
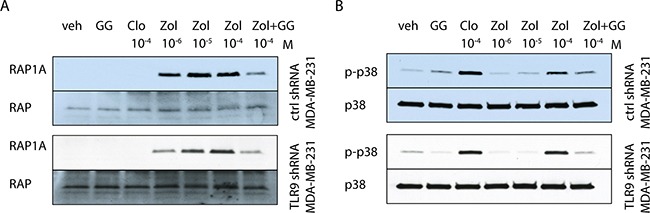
Bisphosphonates have similar effects on p38 and Rap1A in control and TLR9 shRNA cells Western blot images of control and TLR9 shRNA MDA-MB-231 pools. **A**. The cells were treated for 24 h with the indicated bisphosphonates and/or 25 μM geranylgeraniol (GG). Accumulation of unprenylated Rap1A was detected in the cells after 24 h zoledronate (Zol) treatment. This was diminished by simultaneous addition of GG. The same membranes were reblotted with total anti-Rap1 antibody, to demonstrate equal loading. **B**. The cells were also investigated for p38 phosphorylation (p-p38) in response to bisphosphonate-treatment. The blots were reblotted with total anti-p38, to demonstrate equal loading.

To begin to investigate the possible mechanism behind the differences in sensitivity, we investigated BP effects on the mevalonate pathway, using Rap1A prenylation as a marker of n-BP activity [5, 17, 37, 38]. As expected, zoledronate induced a dose-dependent accumulation of unprenylated Rap1A and this was blocked by adding geranylgeraniol to the cultures. The results were similar in both control and TLR9 shRNA MDA-MB-231 cells (Figure 6A). We also compared BP effects on p38 phosphorylation in control and TLR9 shRNA MDA-MB-231 cells. This was done because it has been shown that BP-induced activation of p38 signals for resistance against their growth inhibitory effects [37, 38]. However, both clodronate and zoledronate induced a comparable degree of p38 phosphorylation (Figure 6B). Similar results were seen also with control and TLR9 shRNA T47-C cells (Supplementary Figure S5). Taken together, these results suggest that the increased sensitivity of the TLR9 shRNA cells to the growth inhibitory effects of zoledronate is independent of protein prenylation or p38 phosphorylation.

### MDA-MB-231 TLR9 shRNA cells exhibit increased sensitivity to ApppI

**Figure 7:**
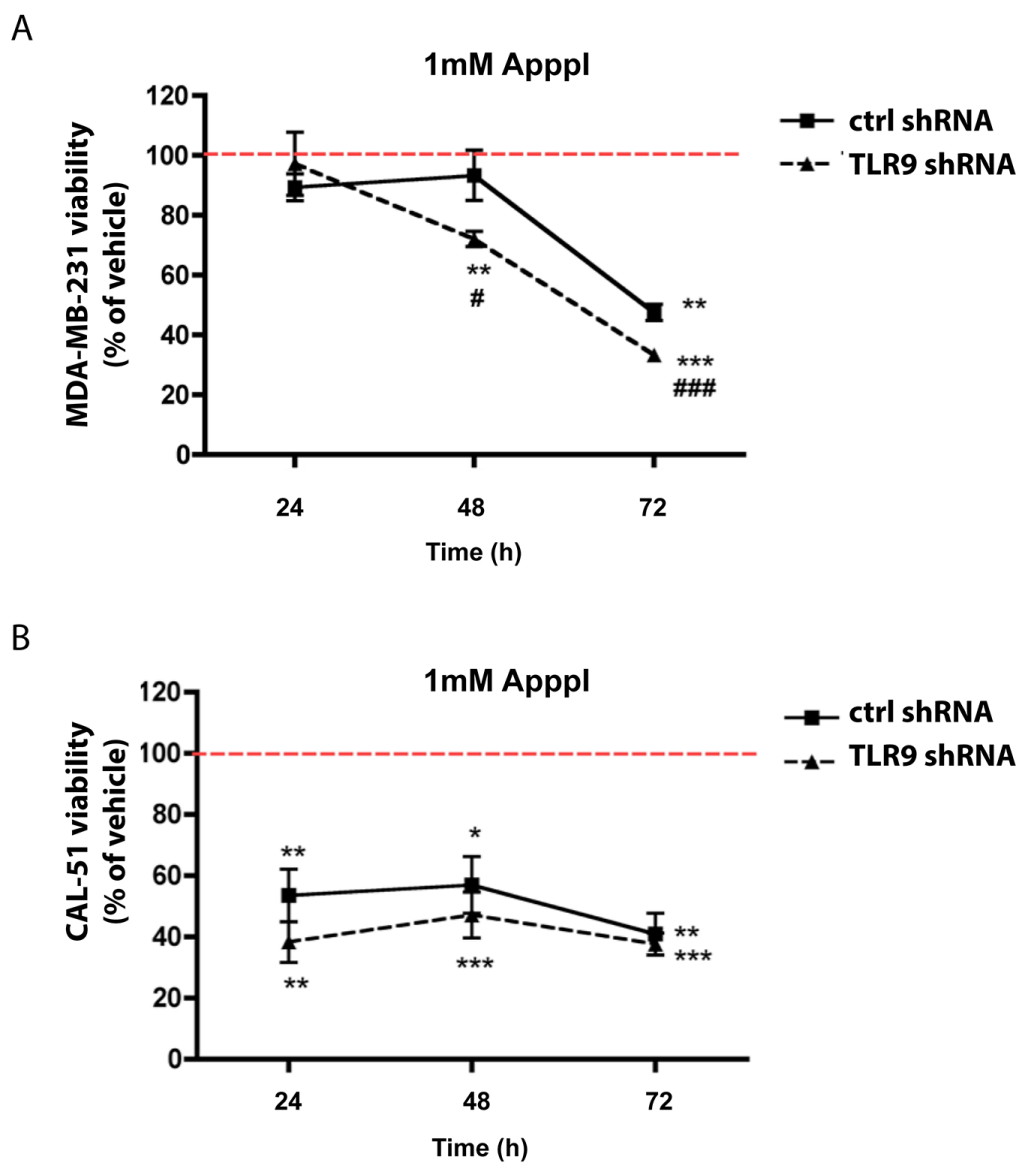
Expression of TLR9 alters growth responses to ApppI **A**. MDA-MB-231 or **B**. CAL-51 control and TLR9 shRNA cells were cultured with 1 mM ApppI for 24 - 72 h. Effects on cell viability was studied with MTS assay. Data is expressed as % of vehicle (indicated with the dotted line). Mean ± s.e.m, n=5-6, * *p*<0.05, ** *p*< 0.01, *** *p*< 0.001 vs. vehicle, # *p* <0.05, ### *p*<0.001 vs. control shRNA cells.

In addition to the prenylation effects, inhibition of the mevalonate pathway by n-BPs also induces the intracellular accumulation of ApppI, which has been suggested to mediate zoledronate-induced cancer cell death [8, 20, 39, 40]. We therefore investigated whether TLR9 expression affects cellular sensitivity to ApppI. Control or TLR9 shRNA cells in the MDA-MB-231 or CAL-51 background were cultured in the presence of vehicle or 1 mM ApppI, followed by assessment of cell viability with MTS assay at various time points. This ApppI concentration was chosen, because it was the lowest of several tested concentrations that demonstrated an effect on the viability of these cells. In general, the CAL-51 cells appeared more sensitive to ApppI, and unlike MDA-MB-231 cells, demonstrated a significant decrease in viability by 1 mM ApppI already after 24 h. Although TLR9 shRNA cells in both backgrounds demonstrated increased sensitivity to ApppI over the corresponding control shRNA cells, this difference was statistically significant only in the MDA-MB-231 background (Figure 7).

### TLR9 shRNA cells exhibit increased sensitivity to the growth inhibitory effects of zoledronate *in vivo*

**Figure 8:**
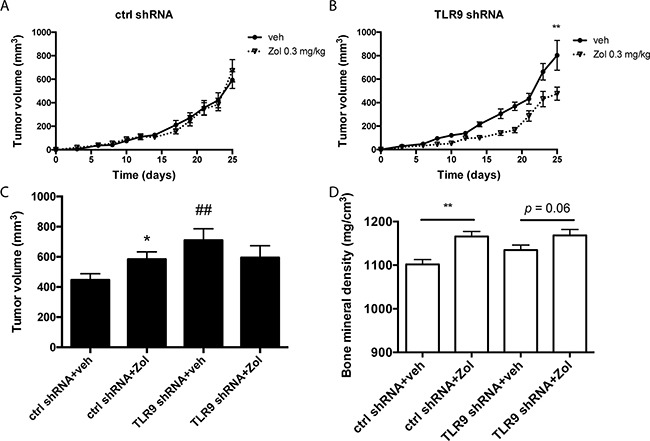
Zoledronate inhibits the growth of MDA-MB-231 TLR9 shRNA cells but not control shRNA cells *in vivo* **A**. Control and **B**. TLR9 shRNA MDA-MB-231 cells were inoculated into the mammary fat pads of nude mice (n = 60, 15 mice per group, 2 tumors per mouse), which were subsequently treated with vehicle or zoledronate. Tumor volumes were measured with a caliper as a function of time, mean ± s.e.m, n=30 (tumors per group), ** *p*<0.01 vs. vehicle-treatment. **C**. Tumor volumes were also measured with caliper after dissection. Data is expressed as mean ± s.e.m, n = 30, * *p*<0.05 vs. vehicle-treatment, ## *p*<0.01 vs. corresponding control shRNA-group. D) Bone mineral densities (BMD) in vehicle- and zoledronate-treated mice were measured with pQCT. Data is expressed as mean ± s.e.m, n = 20 – 21, ** *p*<0.01 vs. corresponding vehicle treatment.

To begin to investigate the *in vivo* significance of our observation, we inoculated control and TLR9 shRNA MDA-MB-231 cells into the mammary fat pads of nude mice, which were subsequently treated with vehicle or zoledronate (0.3 mg/kg 3 times per week, from day 4 to day 25).The aim of this experiment was to establish the proof-of-principle that tumor TLR9 may affect BP responses also *in vivo*. Therefore, to increase the possibility of detecting an anti-tumor effect with zoledronate, the drug was given at a dose and dosing frequency that is higher than those currently given to patients.Tumor growth was followed with caliper measurements as a function of time. We also performed tumor oxygen measurements at day 24, as our previous studies suggested that hypoxia helps to maintain TLR9 expression difference between control and TLR9 shRNA MDA-MB-231 tumors [30]. Our measurements confirmed that all tumors in this study were hypoxic (Supplementary Figure S6). The TLR9 shRNA MDA-MB-231 cells formed larger tumors than the control shRNA cells, as expected based on our previous study [30]. Whereas zoledronate treatment did not affect the growth of the control shRNA tumors, zoledronate significantly inhibited the growth of TLR9 shRNA MDA-MB-231 tumors (Figures 8A – 8B). Furthermore, dissected zoledronate-treated control shRNA tumors had significantly higher volumes than the corresponding vehicle-treated tumors. No such volume difference was detected in dissected TLR9 shRNA tumors (Figure 8C). Finally, we used bone mineral density (BMD) measurements as an intrinsic control for zoledronate efficacy. Zoledronate induced a significant increase of BMD at the cortical bone of tibiae of control shRNA tumor-bearing mice, suggesting that despite the lack of effect on the tumor growth in this group, the drug was biologically active. An increase in BMD was also detected in the zoledronate-treated, TLR9 shRNA tumor-bearing mice (Figure 8D).

## DISCUSSION

Data from a recent meta-analysis support the notion that adjuvant BPs decrease breast cancer mortality and prevent distant recurrence, especially in bone [26]. These beneficial effects were significant only among post-menopausal patients and the absolute benefits remained small. Thus, the numbers needed to treat are high (30 for the mortality benefit and 45 for the bone recurrence benefit) [26]. Furthermore, the cellular and molecular mechanisms for these desired effects are unclear.

We show here, rather surprisingly, that tumor TLR9 expression status affects the direct tumor responses to BPs *in vitro* and *in vivo*. Specifically, low tumor TLR9 expression confers increased cellular sensitivity to the growth inhibitory effects of BPs in breast cancer. Although also detected with clodronate, the effects were most pronounced with n-BPs. We also demonstrated that in some cases BPs actually promoted tumor cell growth and this effect was lost with decreased TLR9 expression. Thus, the increased sensitivity could actually indicate that BPs promote cancer cell growth via TLR9. When TLR9 expression is absent or down-regulated, the growth promotion is lost, resulting in the net effect of tumor inhibition. Although it has been shown also previously that BPs have tumor-promoting effects, they have been largely overlooked midst the reports on their anti-tumor effects. For example, Saarto and coworkers concluded in one of the very first adjuvant BP studies that clodronate increases tumor recurrence at soft tissue sites when given to primary breast cancer patients with node-positive disease [41]. Adjuvant ibandronate significantly increased adrenal metastases in a pre-clinical MDA-MB-231 mouse model [42]. Finally, BPs have demonstrated growth promoting effects in breast cancer cells also *in vitro* [43, 44]. Taken together, our new results suggest that tumor TLR9 expression could mediate these tumor-promoting effects in both ER-positive and ER-negative breast cancer cells. However, no tumor promoting effects by BPs were detected in the recent large meta-analysis of adjuvant BPs, suggesting that in the clinical situation, the net responses of tumors to BPs are either no response or inhibited tumor growth [26].

Interestingly, n-BPs induced a similar accumulation of unprenylated Rap1A in both control and TLR9 shRNA cells, suggesting that the sensitivity difference is independent of n-BP effects on the mevalonate pathway. Furthermore, BPs induced a similar degree of p38 phosphorylation both in control and TLR9 shRNA cells, suggesting that a differential activation of the protective p38-mediated pathway does not explain the results either [18, 37]. Another possibility is that there is redundancy in the activation of these pathways by BPs and thus, these issues require further characterization.

How could TLR9 then mediate cancer cell BP responsiveness? Clues of such mechanisms could be drawn from the inflammatory effects of BPs. In addition to their osteoclast-inhibiting actions, BPs have also well-documented immunomodulatory effects [45]. Specifically, p-BPs are considered anti-inflammatory, while n-BPs are pro-inflammatory [32, 46, 47]. Interestingly, n-BP treatment shares many similarities with activation of inflammasomes, which are cytoplasmic complexes comprised of the ASC, NALP- and Caspase-1 proteins [48]. One of the best understood is the NALP3 inflammasome. Upon ligand binding, NALP3 recruits the inflammatory Caspase-1 into the inflammasome complex. The activated Caspase-1 then processes pro-IL-1β and pro-IL-18 into their active, secreted forms and this alerts the body for inflammation [49]. Also n-BPs induce Caspase-1 activation and IL-1β and IL-18 upregulation [50–54]. This effect of n-BPs could also be mediated via inhibition of the mevalonate pathway as geranylgeraniol, whose formation n-BPs suppress, inhibits Caspase-1 activation. Thus, n-BPs could block the negative feedback of geranylgeraniol on Caspase-1 activation [52, 55]. Our data suggests that TLR9 does not interfere with n-BP effects on geranylgeraniol deprivation. Thus, TLR9 could rather mediate BP effects downstream of Caspase-1 activation. Interestingly, TLR9 has been shown to act in close collaboration with NALP3 inflammasome for example in acetaminophen-induced hepatotoxicity and in acute pancreatitis [56, 57]. Furthermore, adenosine, ATP and ADP have been shown to activate the NALP3 inflammasome [58, 59]. It remains to be studied whether the intracellular ATP-like metabolites of BPs, AppCCl_2_ and ApppI, are also recognized via the NALP3 [8, 39, 40]. TLR9 shRNA cells, however, demonstrated increased sensitivity over that of the control shRNA cells to the growth inhibitory effects of ApppI. Finally, there is also a possible link between the post-menopausal status, tumor TLR9 expression and adjuvant zoledronate anti-tumor efficacy. Menopause has profound effects on both adaptive and innate immune systems. Furthermore, ERα and sex steroids regulate TLR9 expression in breast cancer [28, 60, 61]. Therefore, declining hormone levels could result in decreased TLR9 expression in breast cancer cells. There are no previous publications on menopause effects on TLR9 expression and also these aspects require further investigation.

Our findings have several clinical implications. First, the role of TLR9 as a biomarker for adjuvant BP efficacy should be studied in clinical breast cancer trials. Interestingly, Westbrook and coworkers recently described the first biomarker for BP use in breast cancer. Whether or not TLR9 is on the same molecular pathway than CAPG and GIPC1 remains to be studied [62–63]. Second, we and others recently described a novel, poor prognosis subgroup of breast cancer [30, 63]. These patients have triple-negative disease (TNBC) with low tumor TLR9 expression upon diagnosis. Their disease-specific survival is significantly worse than of those patients whose otherwise similar TNBC tumors have higher TLR9 expression. Especially the low TLR9-TNBC patient group might benefit from adjuvant zoledronate and this should be addressed in a separate clinical trial. Finally, in addition to breast cancer, the significance of our finding should be further studied also in other cancers where BPs are much used or where low tumor TLR9 expression is associated with poor outcome, such as renal cell carcinoma [64].

In conclusion, low tumor TLR9 expression sensitizes breast cancer cells to the growth inhibitory effects of BPs *in vitro* and *in vivo*. Although observed with all BPs, the finding was most pronounced with n-BPs. The molecular mechanisms behind this finding are not known and require further experimenting. The significance of this finding needs to be verified in clinical trials.

## MATERIALS AND METHODS

### Cell culture

Human MDA-MB-231 breast cancer cells and mouse 4T1 mammary tumor cells, which both lack the expression of ER, PR and HER2 and are thus considered as triple-negative, and the estrogen receptor expressing human T47-D and MCF-7 breast cancer cells were originally from ATCC (Manassas, VA) and were cultured as previously described in detail [30]. Human CAL-51 breast cancer cells are also triple-negative and they were purchased from DSMZ (Braunschweig, Germany) and cultured in Dulbecco's modified Eagle's medium (GibcoBRL, Life Technologies, Paisley, UK) supplemented with 10 % heat-inactivated fetal bovine serum, L-glutamine, penicillin/streptomycin, and non-essential amino acids (all from Gibco BRL, Life Technologies) [65]. All cell cultures were done a 37°C atmosphere of 5% CO_2_/95% air (∼ 21 % pO_2_).

### ApppI synthesis

All chemicals for the ApppI synthesis were purchased from Sigma (St. Louis, MO). The detailed procedure for the ApppI synthesis is given in Supplementary Figure 1. Geranylgeraniol (cold, all trans) was purchased from American Radiolabeled Chemicals (St. Louis, MO). Bisphosphonates were purchased from Sigma (St. Louis, MO), dissolved in d-H_2_O at a final concentration of 10^-2^ M, and sterile-filtered.

### RNA interference

TLR9 down-regulation was done with a plasmid-based approach, as previously described. Briefly, TLR9 short hairpin (shRNA) sequence or a control, non-targeting shRNA sequence was cloned into the pSuper vector by the vendor (Oligoengine, Seattle, WA). The plasmids were stably transfected into MDA-MB-231 and single cell clones were selected, using standard techniques. These cells were characterized previously in detail [35]. For stably transfected cell pools (MDA-MB-231 and T47-D), the TLR9 shRNA or control shRNA sequences were first cloned into pSuper-EGFP vector by the vendor (Oligoengine). Transfection, selection and characterization of these cells have been previously published [30]. The CAL-51 and 4T1 cells were transfected with lentiviral-based TLR9 or control shRNA constructs. Characterization and transfection of the CAL-51 cells has been previously described [36]. The murine mammary carcinoma 4T1 cells were stably transfected with mouse-specific lentiviral particles containing either control (non-mammalian) shRNA or TLR9 shRNA (Mission lentiviral transduction particles, Sigma) and selected in the presence of puromycin (6 μg/ml, Sigma). TLR9 mRNA expression in the 4T1 cells was characterized with qRT-PCR, as previously explained [30].

### Western blot analysis

The cells were cultured on 6-well plates in normal culture medium until near confluency, after which they were rinsed with sterile PBS and cultured further for 24 h in serum-free culture medium containing bisphosphonates and/or geranylgeraniol (25 μM). At the desired time-point, the culture medium was discarded and the cells were quickly harvested in lysis buffer (Cell Signaling, Danvers, MA) and clarified by centrifugation, as previously described in detail. After boiling the supernatants in reducing SDS sample buffer, equal amounts of protein (∼100 mg) were loaded per lane and the samples were electrophoresed into 10% or 4-20 % gradient polyacrylamide SDS gels (BioRad, Hercules, CA) and transferred to a nitrocellulose membrane. The membranes were incubated overnight at 4°C with the corresponding antibodies Rap1A SC-1482 (Santa Cruz, Dallas, TX), Rap1 SC-65 (Santa Cruz), p38 (Cell Signaling) or p-p38 (Cell Signaling), diluted 1:500 in Tris-buffered saline, 0.1 % (v/v) Tween-20 (TBST). Secondary detection was performed with HRP-linked secondary antibodies (GE Healthcare, Pittsburgh, PA). The protein bands were visualized by chemiluminescence using ECL kit (Pierce, Rockford, IL) [30, 37].

### Growth assays

For a standard BrdU-assay, 10 000 cells were plated in 100 μl of normal growth medium containing vehicle or the indicated bisphosphonates for an indicated length of time. Incorporated BrdU was measured with a commercial kit, according to the manufacturer's recommendations (Exalpha Biologicals, Watertown, MA). For a standard MTS assay, the cells were plated on 96-well plates (10 000 cells per 100 μl per well) in normal growth medium, supplemented with 1-100 μM of the indicated BPs or vehicle as a control treatment. Cell viability was measured with the CellTiter 96 Aqueus One Solution Cell Proliferation assay (Promega, Madison, Wisconsin), according to the manufacturer's recommendations. Cell growth was also measured as a function of confluency, using IncuCyte FLR and IncuCyte ZOOM® (Essen BioScience Ltd., Hertfordshire, UK) kinetic high-content live cell microscopes. Briefly, 2000-4000 cells were plated in 100 μl per well to 96-well plate with the desired concentrations (1-100 μM) of indicated BPs or vehicle. The default software parameters with a 10× objective were used for imaging. The IncuCyte 2010A or 2014A software (Essen BioScience Ltd.) was used to calculate mean confluences of the individual wells (3-8 parallel wells per treatment group) from phase contrast images.

### *In vivo* experiment

MDA-MB-231 pools, stably transfected with the control or TLR9 shRNA plasmids, were inoculated into the mammary fat pads (10^6^ cells in 100 μl PBS, *n* = 60 mice) of four-week-old female nude mice (Athymic nude/nu Foxn1 mice, Harlan, the Netherlands). Starting on day 3 after tumor cell inoculation, the tumor diameters were measured and tumor volumes were calculated using the formula V = (π/6)(d1 × d2) 3/2, where d1 and d2 are the perpendicular tumor diameters [30]. On day 4, the mice were stratified into 4 groups (*n* = 15 per group), and administered with vehicle or zoledronate (0.3 mg/kg) 3 times a week throughout the experiment. After 3 weeks (on day 26), the mice were sacrificed and the tumors were dissected and measured. Oxygen partial pressures were measured in representative tumors on day 24, as explained below. Throughout the experiments, the animals were maintained under controlled pathogen-free environmental conditions. Animal welfare was monitored daily for clinical signs. The animal experiment procedures were reviewed and approved by the State Provincial Offices of Finland, the license ESAVI/3257/04.10.07/2014.

### Oxygen partial pressure (pO_2_) measurement

In order to measure pO_2_ values in tumors, we used sterile, flexible polarographic electrodes (diameter 0.47 mm) of the Clark type (Licox® GMS, Kiel-Mielkendorf, Germany), supplied with a probe-specific microchip allowing automatic calibration. The probe was inserted into the tumor tissue by advancing it in a retrograde manner along the lumen of an insertion needle catheter, which was then removed. Tissue temperature was measured with a needle probe and temperature-adjusted pO_2_ (mmHg) was graphically displayed and stored digitally. The whole length of the oxygen-sensitive part of the probe was at least 2 mm inside the tumor throughout the measurements to prevent contamination from room-air O_2_. The duration of the pO_2-_measurement was sufficient to establish a stable pO_2_ level, which was then registered and stored. Two tumors from each group at study day 24 were measured over a time period of 20 minutes after a stabilization period of approximately 5 min. The gluteus muscle of the experimental animal served as a control site after measurements to verify the proper function of the Licox^®^ probe (data not shown).

### Bone density measurement

Left tibiae were fixed in 4% formaldehyde, stored in 70% ethanol at 4°C, and scanned using pQCT (XCT 540; Stratec, Birkenfeld, Germany). For the tibiae, 3 cross-sections (at 0.25 mm intervals) were analyzed 1.8 mm from a reference line placed at the proximal end of the tibia, and 1 section was analyzed at the midshaft. Standardized analysis with an image voxel size of 0.07 mm^3^ was performed. Density thresholds of 0.5 mg/cm^3^ for trabecular bone and 0.71 mg/cm^3^ for cortical bone were used.

### Statistical analysis

Data is expressed as mean ± S.D. or ± S.E.M., as indicated. Student's unpaired t-test and one-way ANOVA were used to calculate statistically significant differences (*p*<0.05) between the various study groups.

## SUPPLEMENTARY FIGURES



## Abbreviations

Aln: alendronate
AppCCl_2_: endogenous ATP-analog
ApppI: endogenous ATP-analog
ASC: inflammasome complex protein
BP: bisphosphonate
CAPG: macrophage-capping protein
CI: coincidence interval
Clo: clodronate
ER: estrogen receptor
Eti: etidronate
GIPC1: PDZ domain-containing protein
Her2: receptor tyrosine-protein kinase erbB-2
NALP3: inflammasome complex protein
n-BP: nitrogen-containing bisphosphonate
Pam: pamidronate
p-BP: pyrophosphate-resembling bisphosphonate
PR: progesterone receptor
Ris: risedronate
RR: relative risk
shRNA: short hairpin RNA
TLR9: Toll-like receptor 9
TNBC: triple-negative breast cancer
Zol: zoledronate

## References

[1] Coleman RE, Rathbone E, Brown JE (2013). Management of cancer treatment-induced bone loss. Nat Rev Rheumatol.

[2] Berenson JR, Rosen LS, Howell A, Porter L, Coleman RE, Morley W, Dreicer R, Kuross SA, Lipton A, Seaman JJ (2001). Zoledronic acid reduces skeletal-related events in patients with osteolytic metastases. Cancer.

[3] Jimenez-Soriano Y, Bagan JV (2005). Bisphosphonates, as a new cause of drug-induced jaw osteonecrosis: an update. Med Oral Patol Oral Cir Bucal.

[4] Gordon DH (2005). Efficacy and safety of intravenous bisphosphonates for patients with breast cancer metastatic to bone: a review of randomized, double-blind, phase III trials. Clin Breast Cancer.

[5] Rogers MJ (2004). From molds and macrophages to mevalonate: a decade of progress in understanding the molecular mode of action of bisphosphonates. Calcif Tissue Int.

[6] Lehenkari PP, Kellinsalmi M, Näpänkangas JP, Ylitalo KV, Mönkkönen J, Rogers MJ, Azhayev A, Väänänen HK, Hassinen IE (2002). Further insight into mechanism of action of clodronate: inhibition of mitochondrial ADP/ATP translocase by a nonhydrolyzable, adeninecontaining metabolite. Mol Pharmacol.

[7] Alakangas A, Selander K, Mulari M, Halleen J, Lehenkari P, Mönkkönen J, Salo J, Väänänen K (2002). Alendronate disturbs vesicular trafficking in osteoclasts. Calcif Tissue Int.

[8] Mönkkönen H, Auriola S, Lehenkari P, Kellinsalmi M, Hassinen IE, Vepsäläinen J, Mönkkönen J (2006). A new endogenous ATP analog (ApppI) inhibits the mitochondrial adenine nucleotide translocase (ANT) and is responsible for the apoptosis induced by nitrogen-containing bisphosphonates. Br J Pharmacol.

[9] Cremers SC, Pillai G, Papapoulos SE (2005). Pharmacokinetics/pharmacodynamics of bisphosphonates: use for optimisation of intermittent therapy for osteoporosis. Clin Pharmacokinet.

[10] Brown JE, Neville-Webbe H, Coleman RE (2004). The role of bisphosphonates in breast and prostate cancers. Endocr Relat Cancer.

[11] Croucher P, Jagdev S, Coleman R (2003). The anti-tumor potential of zoledronic acid. Breast.

[12] Daubiné F, Le Gall C, Gasser J, Green J, Clézardin P (2007). Antitumor effects of clinical dosing regimens of bisphosphonates in experimental breast cancer bone metastasis. J Natl Cancer Inst.

[13] Dumon JC, Journé F, Kheddoumi N, Lagneaux L, Body JJ (2004). Cytostatic and apoptotic effects of bisphosphonates on prostate cancer cells. Eur Urol.

[14] Fromigue O, Lagneaux L, Body JJ (2000). Bisphosphonates induce breast cancer cell death in vitro. J Bone Miner Res.

[15] Hiraga T, Williams PJ, Ueda A, Tamura D, Yoneda T (2004). Zoledronic acid inhibits visceral metastases in the 4T1/luc mouse breast cancer model. Clin Cancer Res.

[16] Tuomela JM, Valta MP, Väänänen K, Härkönen PL (2008). Alendronate decreases orthotopic PC-3 prostate tumor growth and metastasis to prostate-draining lymph nodes in nude mice. BMC Cancer.

[17] Wakchoure S, Merrell MA, Aldrich W, Millender-Swain T, Harris KW, Triozzi P, Selander KS (2006). Bisphosphonates inhibit the growth of mesothelioma cells in vitro and in vivo. Clin Cancer Res.

[18] Merrell MA, Wakchoure S, Lehenkari PP, Harris KW, Selander KS (2007). Inhibition of the mevalonate pathway and activation of p38 MAP kinase are independently regulated by nitrogen-containing bisphosphonates in breast cancer cells. Eur J Pharmacol.

[19] Shipman CM, Croucher PI, Russell RG, Helfrich MH, Rogers MJ (1998). The bisphosphonate incadronate (YM175) causes apoptosis of human myeloma cells in vitro by inhibiting the mevalonate pathway. Cancer Res.

[20] Mitrofan LM, Pelkonen J, Mönkkönen J (2009). The level of ATP analog and isopentenyl pyrophosphate correlates with zoledronic acid-induced apoptosis in cancer cells in vitro. Bone.

[21] Coleman RE (2009). Adjuvant bisphosphonates in breast cancer: are we witnessing the emergence of a new therapeutic strategy?. Eur J Cancer.

[22] Diel IJ, Mundy GR, Bone International, Cancer Study Group IBCG (2000). Bisphosphonates in the adjuvant treatment of cancer: experimental evidence and first clinical results. Br J Cancer.

[23] Coleman R, Cameron D, Dodwell D, Bell R, Wilson C, Rathbone E, Keane M, Gil M, Burkinshaw R, Grieve R, Barrett-Lee P, Ritchie D, Liversedge V, AZURE investigators (2014). Adjuvant zoledronic acid in patients with early breast cancer: final efficacy analysis of the AZURE (BIG 01/04) randomised open-label phase 3 trial. Lancet Oncol.

[24] Gnant M, Mlineritsch B, Stoeger H, Luschin-Ebengreuth G, Knauer M, Moik M, Jakesz R, Seifert M, Taucher S, Bjelic-Radisic V, Balic M, Eidtmann H, Eiermann W (2015). and Austrian Breast and Colorectal Cancer Study Group, Vienna, Austria. Zoledronic acid combined with adjuvant endocrine therapy of tamoxifen versus anastrozol plus ovarian function suppression in premenopausal early breast cancer: final analysis of the Austrian Breast and Colorectal Cancer Study Group Trial 12. Ann Oncol.

[25] He M, Fan W, Zhang X (2013). Adjuvant zoledronic acid therapy for patients with early stage breast cancer: an updated systematic review and meta-analysis. J Hematol Oncol.

[26] Coleman R, Powles T, Paterson A, Gnant M, Anderson S, Diel I, Gralow J, von Minckwitz G, Moebus V, Bergh J, Pritchard KI, Bliss J, Cameron D (2015). and Early Breast Cancer Trialists’ Collaborative Group (EBCTCG). Adjuvant bisphosphonate treatment in early breast cancer: meta-analyses of individual patient data from randomised trials. Lancet.

[27] Hemmi H, Takeuchi O, Kawai T, Kaisho T, Sato S, Sanjo H, Matsumoto M, Hoshino K, Wagner H, Takeda K, Akira S (2000). A Toll-like receptor recognizes bacterial DNA. Nature.

[28] Jukkola-Vuorinen A, Rahko E, Vuopala KS, Desmond R, Lehenkari PP, Harris KW, Selander KS (2009). Toll-like receptor-9 expression is inversely correlated with estrogen receptor status in breast cancer. J Innate Immun.

[29] Merrell MA, Ilvesaro JM, Lehtonen N, Sorsa T, Gehrs B, Rosenthal E, Chen D, Shackley B, Harris KW, Selander KS (2006). Toll-like receptor 9 agonists promote cellular invasion by increasing matrix metalloproteinase activity. Mol Cancer Res.

[30] Tuomela J, Sandholm J, Karihtala P, Ilvesaro J, Vuopala KS, Kauppila JH, Kauppila S, Chen D, Pressey C, Härkönen P, Harris KW, Graves D, Auvinen PK (2012). Low TLR9 expression defines an aggressive subtype of triple-negative breast cancer. Breast Cancer Res Treat.

[31] Dicuonzo G, Vincenzi B, Santini D, Avvisati G, Rocci L, Battistoni F, Gavasci M, Borzomati D, Coppola R, Tonini G (2003). Fever after zoledronic acid administration is due to increase in TNF-alpha and IL-6. J Interferon Cytokine Res.

[32] Funayama H, Ohsako M, Monma Y, Mayanagi H, Sugawara S, Endo Y (2005). Inhibition of inflammatory and boneresorption-inhibitory effects of alendronate by etidronate. Calcif Tissue Int.

[33] Richards PJ, Amos N, Williams AS, Williams BD (1999). Pro-inflammatory effects of the aminobisphosphonate ibandronate in vitro and in vivo. Rheumatology (Oxford).

[34] Norton JT, Hayashi T, Crain B, Corr M, Carson DA (2011). Role of IL-1 receptor-associated kinase-M (IRAK-M) in priming of immune and inflammatory responses by nitrogen bisphosphonates. Proc Natl Acad Sci USA.

[35] Ilvesaro JM, Merrell MA, Li L, Wakchoure S, Graves D, Brooks S, Rahko E, Jukkola-Vuorinen A, Vuopala KS, Harris KW, Selander KS (2008). Toll-like receptor 9 mediates CpG oligonucleotide-induced cellular invasion. Mol Cancer Res.

[36] Tuomela JM, Sandholm JA, Kaakinen M, Hayden KL, Haapasaari KM, Jukkola-Vuorinen A, Kauppila JH, Lehenkari PP, Harris KW, Graves DE, Selander KS (2016). Telomeric G-quadruplex-forming DNA fragments induce TLR9-mediated and LL-37-regulated invasion in breast cancer cells in vitro. Breast Cancer Res Treat.

[37] Merrell M, Suarez-Cuervo C, Harris KW, Väänänen HK, Selander KS (2003). Bisphosphonate induced growth inhibition of breast cancer cells is augmented by p38 inhibition. Breast Cancer Res Treat.

[38] Dunford JE, Rogers MJ, Ebetino FH, Phipps RJ, Coxon FP (2006). Inhibition of protein prenylation by bisphosphonates causes sustained activation of Rac, Cdc42, and Rho GTPases. J Bone Miner Res.

[39] Mönkkönen H, Ottewell PD, Kuokkanen J, Mönkkönen J, Auriola S, Holen I (2007). Zoledronic acid-induced IPP/ApppI production in vivo. Life Sci.

[40] Jauhiainen M, Mönkkönen H, Räikkönen J, Mönkkönen J, Auriola S (2009). Analysis of endogenous ATP analogs and mevalonate pathway metabolites in cancer cell cultures using liquid chromatography-electrospray ionization mass spectrometry. J Chromatogr B Analyt Technol Biomed Life Sci.

[41] Saarto T, Blomqvist C, Virkkunen P, Elomaa I (2001). Adjuvant clodronate treatment does not reduce the frequency of skeletal metastases in node-positive breast cancer patients: 5-year results of a randomized controlled trial. J Clin Oncol.

[42] Michigami T, Hiraga T, Williams PJ, Niewolna M, Nishimura R, Mundy GR, Yoneda T (2002). The effect of the bisphosphonate ibandronate on breast cancer metastasis to visceral organs. Breast Cancer Res Treat.

[43] Journe F, Body JJ, Leclercq G, Nonclercq D, Laurent G (2004). Estrogen responsiveness of IBEP-2, a new human cell line derived from breast carcinoma. Breast Cancer Res Treat.

[44] Journe F, Chaboteaux C, Dumon JC, Leclercq G, Laurent G, Body JJ (2004). Steroid-free medium discloses oestrogenic effects of the bisphosphonate clodronate on breast cancer cells. Br J Cancer.

[45] Rogers MJ, Crockett JC, Coxon FP, Monkkonen J (2011). Biochemical and molecular mechanisms of action of bisphosphonates. Bone.

[46] Monkkonen J, Simila J, Rogers MJ (1998). Effects of tiludronate and ibandronate on the secretion of proinflammatory cytokines and nitric oxide from macrophages in vitro. Life Sci.

[47] Makkonen N, Salminen A, Rogers MJ, Frith JC, Urtti A, Azhayeva E, Monkkonen J (1999). Contrasting effects of alendronate and clodronate on RAW 264 macrophages: the role of a bisphosphonate metabolite. Eur J Pharm Sci.

[48] Man SM, Kanneganti TD (2015). Regulation of inflammasome activation. Immunol Rev.

[49] Martinon F (2008). Detection of immune danger signals by NALP3. J Leukoc Biol.

[50] Deng X, Tamai R, Endo Y, Kiyoura Y (2009). Alendronate augments interleukin-1beta release from macrophages infected with periodontal pathogenic bacteria through activation of caspase-1. Toxicol Appl Pharmacol.

[51] Maugeri D, Mamazza C, Lo Giudice F, Puglisi N, Muscoso EG, Rizzotto M, Testai M, Bennati E, Lentini A, Panebianco P (2005). Interleukin-18 (IL-18) and matrix metalloproteinase-9 (MMP-9) in post-menopausal osteoporosis. Arch Gerontol Geriatr.

[52] Benford HL, Frith JC, Auriola S, Monkkonen J, Rogers MJ (1999). Farnesol and geranylgeraniol prevent activation of caspases by aminobisphosphonates: biochemical evidence for two distinct pharmacological classes of bisphosphonate drugs. Mol Pharmacol.

[53] Shikama Y, Nagai Y, Okada S, Oizumi T, Shimauchi H, Sugawara S, Endo Y (2010). Pro-IL-1β accumulation in macrophages by alendronate and its prevention by clodronate. Toxicol Lett.

[54] Benford HL, McGowan NW, Helfrich MH, Nuttall ME, Rogers MJ (2001). Visualization of bisphosphonate-induced caspase-3 activity in apoptotic osteoclasts in vitro. Bone.

[55] Montero MT, Matilla J, Gomez-Mampaso E, Lasuncion MA (2004). Geranylgeraniol regulates negatively caspase-1 autoprocessing: implication in the Th1 response against Mycobacterium tuberculosis. J Immunol.

[56] Imaeda AB, Watanabe A, Sohail MA, Mahmood S, Mohamadnejad M, Sutterwala FS, Flavell RA, Mehal WZ (2009). Acetaminophen-induced hepatotoxicity in mice is dependent on Tlr9 and the Nalp3 inflammasome. J Clin Invest.

[57] Hoque R, Sohail M, Malik A, Sarwar S, Luo Y, Shah A, Barrat F, Flavell R, Gorelick F, Husain S, Mehal W (2011). TLR9 and the NLRP3 inflammasome link acinar cell death with inflammation in acute pancreatitis. Gastroenterology.

[58] Baron L, Gombault A, Fanny M, Villeret B, Savigny F, Guillou N, Panek C, Le Bert M, Lagente V, Rassendren F, Riteau N, Couillin I (2015). The NLRP3 inflammasome is activated by nanoparticles through ATP, ADP and adenosine. Cell Death Dis.

[59] Gicquel T, Victoni T, Fautrel A, Robert S, Gleonnec F, Guezingar M, Couillin I, Catros V, Boichot E, Lagente V (2014). Involvement of purinergic receptors and NOD-like receptor-family protein 3-inflammasome pathway in the adenosine triphosphate-induced cytokine release from macrophages. Clin Exp Pharmacol Physiol.

[60] Sandholm J, Kauppila JH, Pressey C, Tuomela J, Jukkola-Vuorinen A, Vaarala M, Johnson MR, Harris KW, Selander KS (2012). Estrogen receptor-α and sex steroid hormones regulate Toll-like receptor-9 expression and invasive function in human breast cancer cells. Breast Cancer Res Treat.

[61] Giefing-Kroll C, Berger P, Lepperdinger G, Grubeck-Loebenstein B (2015). How sex and age affect immune responses, susceptibility to infections, and response to vaccination. Aging Cell.

[62] Westbrook JA, Cairns DA, Peng J, Speirs V, Hanby AM, Holen I, Wood SL, Ottewell PD, Marshall H, Banks RE, Selby PJ, Coleman RE, Brown JE (2016). CAPG and GIPC1: Breast Cancer Biomarkers for Bone Metastasis Development and Treatment. J Natl Cancer Inst.

[63] Meseure D, Vacher S, Drak Alsibai K, Trassard M, Nicolas A, Leclere R, Lerebours F, Guinebretiere JM, Marangoni E, Lidereau R, Bieche I (2016). Biopathological Significance of TLR9 Expression in Cancer Cells and Tumor Microenvironment Across Invasive Breast Carcinomas Subtypes. Cancer Microenviron.

[64] Ronkainen H, Hirvikoski P, Kauppila S, Vuopala KS, Paavonen TK, Selander KS, Vaarala MH (2011). Absent Tolllike receptor-9 expression predicts poor prognosis in renal cell carcinoma. J Exp Clin Cancer Res.

[65] Lehmann BD, Bauer JA, Chen X, Sanders ME, Chakravarthy AB, Shyr Y, Pietenpol JA (2011). Identification of human triple-negative breast cancer subtypes and preclinical models for selection of targeted therapies. J Clin Invest.

