# Supplementary material for: Toll-like receptor 9 expression is associated with breast cancer sensitivity to the growth inhibitory effects of bisphosphonates in vitro and in vivo
Source: Oncotarget. 2016 Nov 24;7(52):87373–89. doi: 10.18632/oncotarget.13570 (PMC5349995; doi:10.18632/oncotarget.13570)
Supplement: Supplementary file 1 [file oncotarget-07-87373-s001.pdf]

# Toll-like receptor 9 expression is associated with breast cancer sensitivity to the growth inhibitory effects of bisphosphonates *in vitro* and *in vivo*

## SUPPLEMENTARY FIGURES

Synthesis of Apppl.

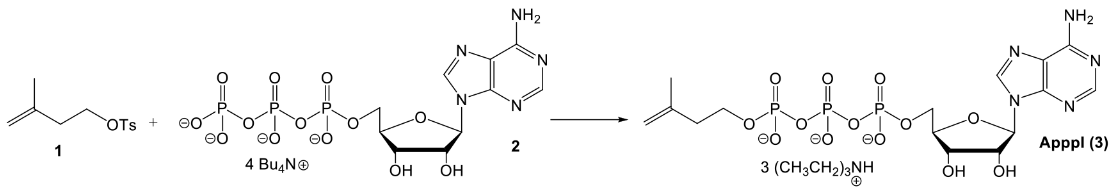


**Supplementary Figure S1: Synthesis of ApppI was carried out in steps 1-3, as outlined in Figure 1. Isopentenyl Tosylate (step 1):** A solution of isopentenyl alcohol (2.0 g, 2.4 mL, 23.2 mmol) in anhydrous pyridine (3.8 mL, 46.4 mmol) was placed in an ice bath at 0°C and stirred for 10 minutes. *p*-Toluene sulfonyl chloride (4.4 g, 23.2 mmol) was added and the mixture was allowed to stir for an additional 15 minutes in the ice bath. After removing from the ice bath, stirring was continued for an additional 4 h at room temperature. The reaction mixture was diluted with water (50 mL) and extracted with $Et_2O$ (3 × 50 mL). The combined organic layers were washed with 0.05 M $H_2SO_4$ (1 × 20 mL), brine (1 × 50 mL), dried ($Na_2SO_4$), filtered and concentrated to dryness *in vacuo* to give a colorless oil. The crude product was purified by flash column chromatography over Si gel using 5-10% $Et_2O$ in hexanes as eluent to afford compound **1** as a colorless oil (3.8 g, 68 %); $^1$H NMR (300 MHz, $CDCl_3$): d 1.67 (s, 3H), 2.36 (t, 2H, $J$ = 7.2 Hz), 2.46 (s, 3H), 4.13 (t, 2H, $J$ = 7.2 Hz), 4.68 (s, 1H), 4.79 (s, 1H), (7.35 (d, 2H, $J$ = 8.0 Hz), 7.80 (d, 2H, $J$ = 8.0 Hz); $^{13}$C NMR (75 MHz, $CDCl_3$): d 21.8, 22.5, 36.9, 68.7, 113.3, 128.1 (2C), 129.9, 140.3 and 145.0. **ATP tetrabutyl ammonium salt (Step 2):** ATP disodium salt (1 mmol) was converted into its acidic form by treatment of Dowex 50WX8-100-200 ($H^+$) ion exchange resin and the eluent was promptly titrated with $n$$Bu_4$NOH to pH 8. The first 150 mL or so of eluent was collected and concentrated to 15 mL under high vacuum and the reduced volume was lyophilized for 48 h to give the tetrabutyl ammonium salt of ATP **(2)** as a foamy, hygroscopic solid. This product was used for the next reaction without further purification. **Triphosphoric acid 1-adenosin-50-yl ester 3-(3-methylbut-3-enyl) ester (ApppI, Step 3):** Compound **2** (0.67 g, 0.46 mmol) was dissolved in anhydrous$CH_3CN$ (8 mL) and treated with isopentenyl tosylate **1** (0.11 g, 0.46 mmol) with stirring under $N_2$. The solution was stirred at room temperature overnight and then concentrated to dryness *in vacuo*. The oily residue was dissolved in a mixture of water (5 mL) and $CH_3CN$ (1 mL) and purified by semi-preparative HPLC using 0.1M TEAB in $CH_3CN$ as eluent. The fractions containing pure product were combined and concentrated to dryness under high vacuum. The residue obtained was dissolved in water (10 mL) and lyophilized for 48 h to afford triethyl ammonium salt form of **ApppI (3)** as a white, amorphous, hygroscopic solid (0.12 g, 30.1%). $^1$H NMR (400 MHz, $D_2O$): d 1.23 (t, 27H, $J$ = 7.3 Hz), 1.62 (s, 3H), 2.23 (t, 2H, $J$ = 6.7 Hz), 3.12 (q, 18H), 3.88-4.03 (m, 2H), 4.11-4.29 (m, 2H), 4.31-4.40 (m, 1H), 4.5-4.55 (m, 1H), 4.69-4.72 (m, 2H), 4.76-4.82 (m, 1H), 6.11 (d, 1H, $J$ = 5.9 Hz), 8.24 (s, 1H), 8.53 (s, 1H) and MS (ESI) *m*/*z*: 576 [M+H]. The final product was dissolved in sterile d-H2O, aliquoted and stored at -20°C.

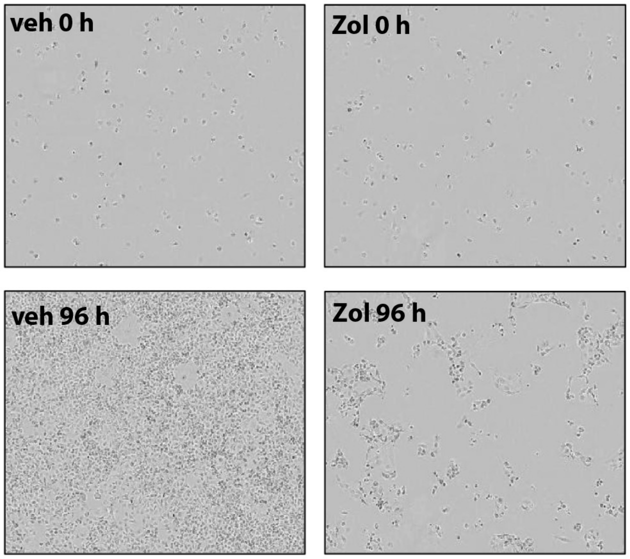


**Supplementary Figure S2: Representative images from 96-well plates onto which the various cell lines were seeded.** The images were taken with the IncuCyte set-up, and they represent the images from which the confluencies of the indicated cells were calculated. Time points 0 h and 96 h are shown.

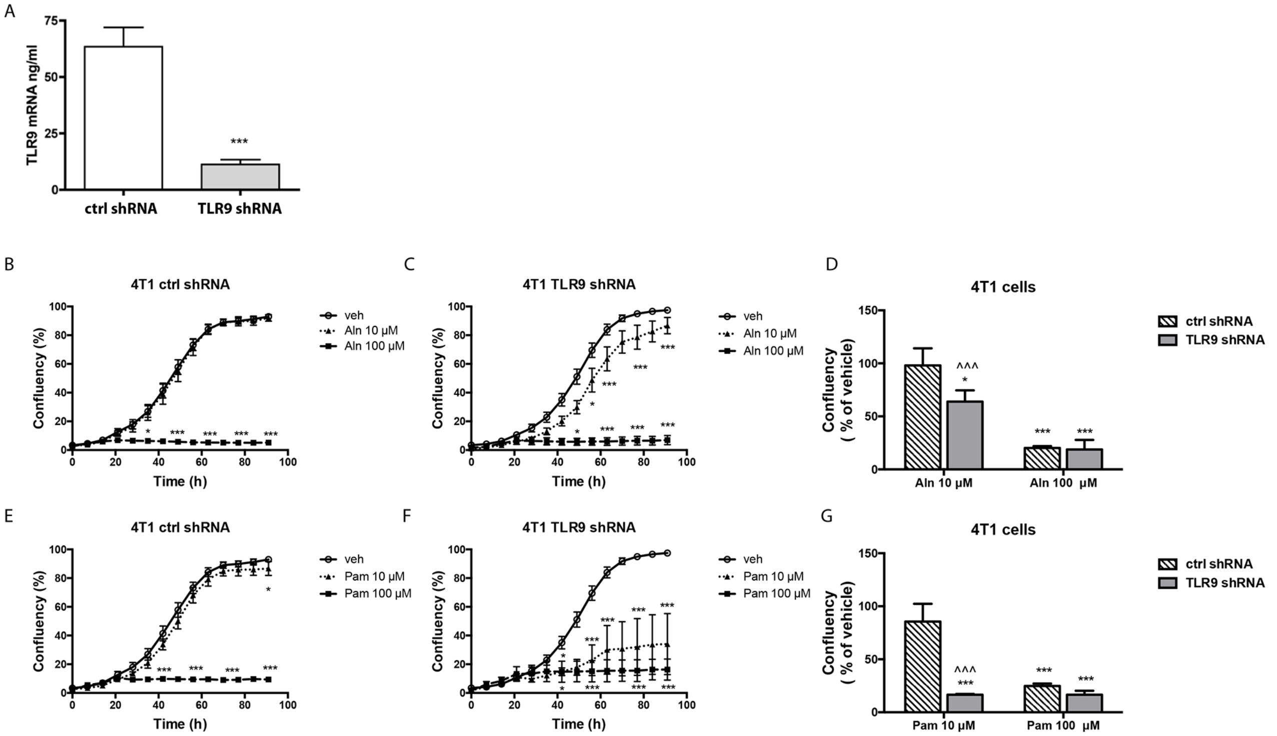


**Supplementary Figure S3: A.** TLR9 mRNA expression in mouse mammary cancer 4T1 cells, stably transfected with lentiviral control shRNA or TLR9 shRNA. Data is expressed as mean ± s.e.m, n = 9, *** p <0.001 vs. control siRNA cells. The control shRNA (**B, E**) or TLR9 shRNa (**C, F**) were cultured in the presence of indicated concentrations of alendronate (**B - D**) or pamidronate (**E - G**). Cell growth was measured as a function of confluency with the IncuCyte image analysis system. % confluency compared to vehicle was estimated at the final time point and compared between control and TLR9 shRNA cells (d, g). Data is expressed as mean ± s.e.m (**B, C, E, F**) or mean ± s.d (D, G), n= 4. * p<0.05, ** p<0.01, *** p<0.001 vs. vehicle, ^^^ p< 0.001 vs. corresponding control shRNA cells.

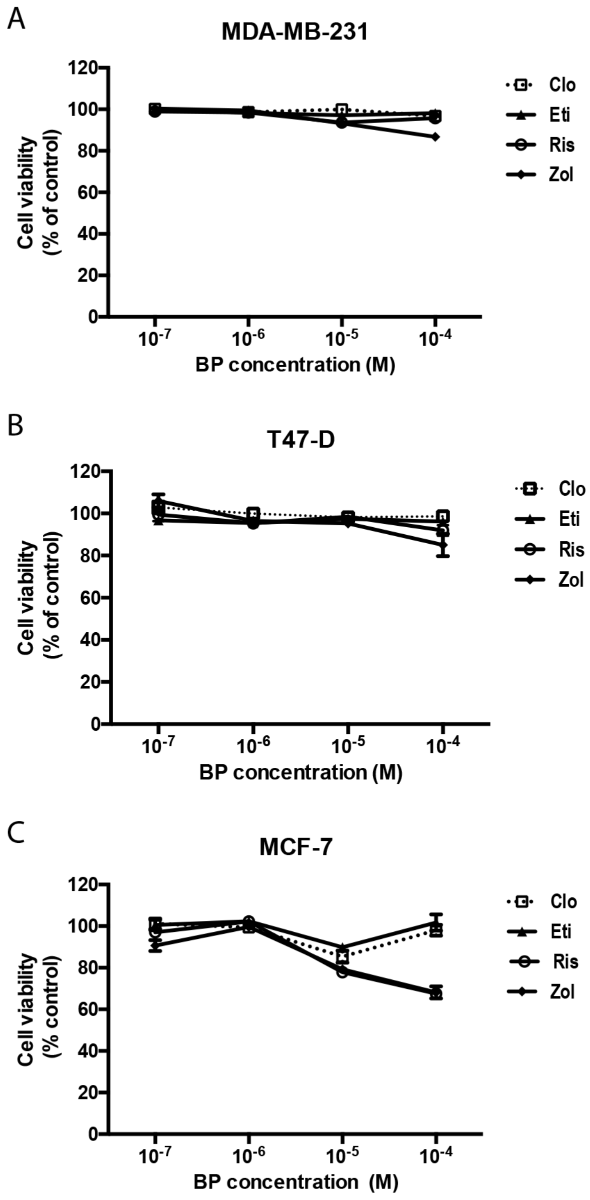


**Supplementary Figure S4: A.** MDA-MB-231, **B.** T47-D and **C.** MCF-7 cells were plated on 96-well plates with vehicle or indicated BPs. Cellular viability was measured 24 h later with MTS assay. Data is expressed as viability % of vehicle, mean ± s.e.m, n=9-12. MCF-7 cells were significantly more sensitive to the growth inhibitory effects of all studied bisphosphonates, as compared with their effects on MDA-MB-231 and T47-D cells. These effects were, however, slightly dependent on the BP and concentration used as follows. MCF-7 cells vs. MDA-MB-231 cells, comparison between % cell viabilities with treatments; clodronate $10^{-5}$ M $p<0.001$, etidronate $10^{-5}$ M $p<0.05$, risedronate $10^{-4}$ M and $10^{-5}$ M $p<0.001$, zoledronate $10^{-4}$, $10^{-5}$ and $10^{-7}$ M $p<0.001$. MCF-7 cells vs. T47-D cells, comparison between % cell viabilities with treatments; clodronate $10^{-5}$ M $p<0.05$, etidronate $10^{-4}$ and $10^{-5}$ M $p<0.05$, risedronate $10^{-4}$M and $10^{-5}$ M $p<0.001$, risedronate $10^{-6}$ M $p<0.05$, zoledronate $10^{-4}$, $10^{-5}$ and $10^{-7}$ M $p<0.001$.

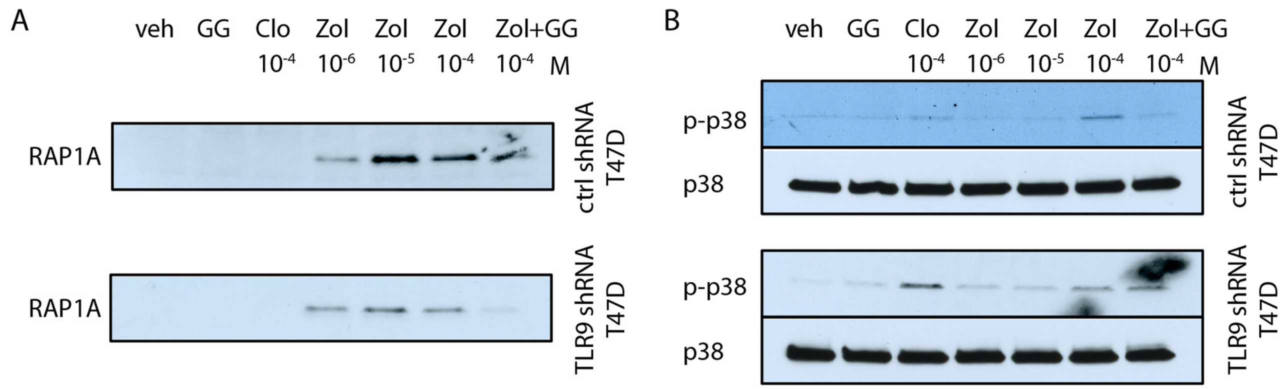


**Supplementary Figure S5: Similar effects of bisphosphonates on p38 and Rap1A in control and TLR9 shRNA cells.** Western blot images of control and TLR9 shRNA T47-D pools. **A.** The cells were treated for 24 h with the indicated bisphosphonates and/or 25 µM geranylgeraniol (GG). Accumulation of unprenylated Rap1A was detected in the cells after 24 h bisphosphonate treatment. This was diminished by simultaneous addition of 25 µM GG. **B.** The same blots were stripped and blotted with anti-phospho-p38 (p-p38) and total anti-p38 antibodies, to investigate BP effects on p38 phosphorylation in response to bisphosphonate treatment.

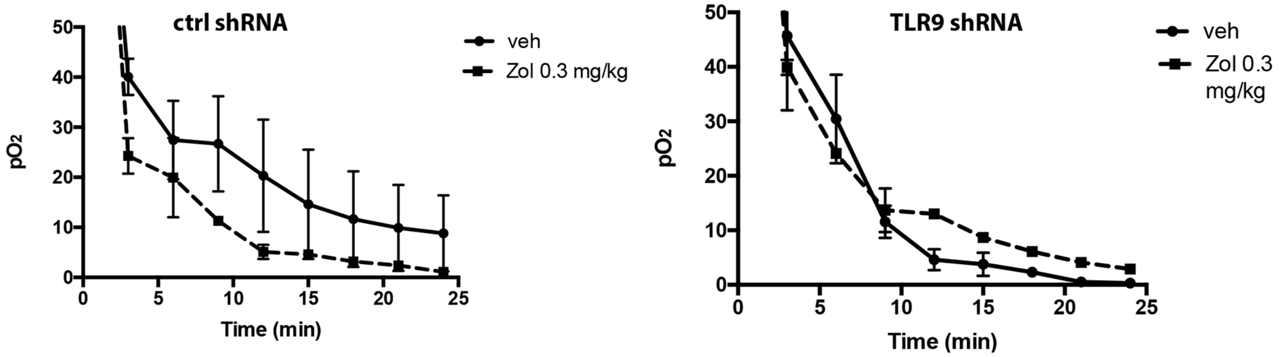


**Supplementary Figure S6: Tissue oxygen measurement with Licox® polarographic catheter from in vivo xenograft tumors.** Hypoxia was documented in all tumors (n=3).
